# Blastomycosis‐like pyoderma in an immunocompetent patient

**DOI:** 10.1002/ccr3.5479

**Published:** 2022-02-25

**Authors:** Fatma Sahli, Noureddine Litaiem, Soumaya Gara, Olfa Charfi, Soumaya Rammeh, Faten Zeglaoui

**Affiliations:** ^1^ 37960 Department of Dermatology Charles Nicolle Hospital Tunis Tunisia; ^2^ Faculty of Medicine of Tunis University of Tunis El Manar Tunis Tunisia; ^3^ 37960 Department of Pathology Charles Nicolle Hospital Tunis Tunisia

**Keywords:** dermatology, health maintenance, infectious diseases

## Abstract

Blastomycosis‐like pyoderma is a rare skin disorder most commonly caused by bacterial infection. It is usually diagnosed in immunocompromised patients. We report a case of BLP in an immunocompetent woman, who presented with a 6‐week history of verrucous cutaneous plaque of the left wrist.

## Graphical abstract

BLP is a rare and uncommon inflammatory disease of the skin. The diagnosis is sometimes confusing, and the clinical aspect may suggest pyoderma gangrenosum, mycobacterial infection, or cutaneous tuberculosis.

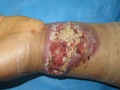

## 1 INTRODUCTION

Blastomycosis‐like pyoderma (BLP) is a rare cutaneous disorder. It presents as large verrucous plaques with multiple pustules and an elevated border.^1^ It is considered to be an exaggerated inflammatory response to bacteria, especially *Staphylococcus aureus*.

Blastomycosis‐like pyoderma often occurs in patients with impaired immunological capacity.^2^ We describe a rare case of BLP in an immunocompetent female patient.

## 2 CASE REPORT

**FIGURE 1:**
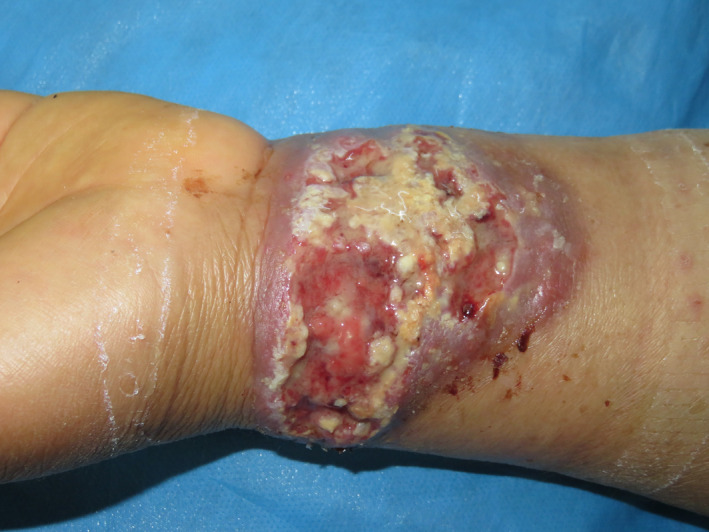
Vegetating plaque of the right wrist with elevated borders, multiple pustules, and central ulceration

A 65‐year‐old woman with type 2 diabetes mellitus was presented with a 6‐week history of verrucous cutaneous plaque of the anterior aspect of the left wrist. The lesion was painful and ulcerated. The patient denied any history of trauma or contact with patients with tuberculosis. Dermatological examination revealed an irregular, erythematous, and vegetating plaque of the right wrist, measuring 10 cm × 4 cm, with elevated and erythematous borders with multiple pustules of different sizes and central ulceration (Figure 1). There was no palpable lymphadenopathy, and physical examination was otherwise unremarkable.

Bacterial culture from a pus swab identified *Staphylococcus aureus*. Histopathological examination of a skin biopsy specimen showed acanthosis of the epidermis with inflammatory infiltration of the dermis made of lymphocytes, neutrophils, plasma cells, and histiocytes. The search of leishmania by direct microscopy and polymerase chain reaction was negative.

**FIGURE 2:**
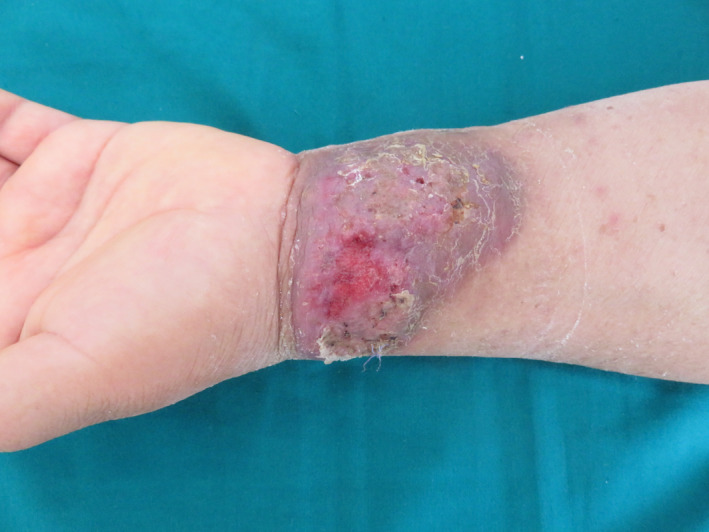
Substantial improvement after 6‐day antibiotic treatment

**FIGURE 3:**
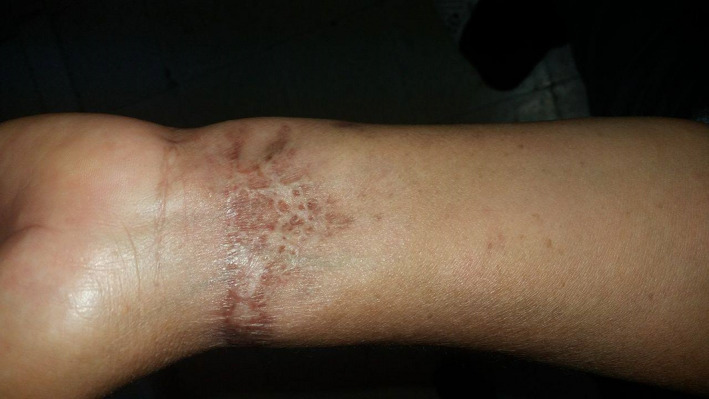
A resolution was noted after 6 months leaving a dyspigmented scar

The diagnosis of BLP was made. The patient received amoxicillin (3 g daily) with clavulanic acid (62.5 mg daily) and local wound care for 20 days. Substantial improvement was seen after 6 days (Figure 2). A total resolution was obtained, and no recurrence was noted after 6 months (Figure 3).

## 3 DISCUSSION

Blastomycosis‐like pyoderma is an exaggerated chronic inflammatory reaction due to a bacterial infection.^1^ It presents as vegetating skin lesions similar to warty tuberculosis and blastomycosis.^2^ The latter could, however, have several clinical presentations. BLP is usually diagnosed in immunocompromised patients.^2^ In these cases, BLP is related to several factors: HIV infection, alcoholism, neoplasia, malnutrition, and immunosuppressant drugs.^2^ Cases of BLP in immunocompetent patients are rare. Local factors may be involved including injury, foreign bodies, tattoos, radiotherapy, and trauma.^3^

Numerous microorganisms are associated with BLP including *Staphylococcus*, *Streptococcus pyogenes*, *Pseudomonas aeruginosa*, *Escherichia coli*, and *Candida albicans*. However, *Staphylococcus aureus* is the most common causative microorganism. The mechanism by which these microorganisms induce PLP remains unknown. Many believe that the microbiological agent is not directly responsible for the disease but by creating an immune dysfunction.^3^

Su et al. introduced^2^ diagnostic criteria for BLP: (i) large verrucous plaques with multiple pustules and elevated border, (ii) histological evidence of pseudoepitheliomatous hyperplasia with abscesses, (iii) identifications of bacteria by tissue culture, (iv) negative culture for fungi and mycobacteria, (v) negative fungal serology tests, and (vi) normal bromide and iodide blood levels. Our patient met four of these criteria.

Differential diagnoses include pyoderma gangrenosum, pemphigus vegetans, vegetating iododerma, chromomycosis, and sporotrichosis.^4^ In contrast to blastomycosis‐like pyoderma, pyoderma gangrenosum is characterized by a sterile inflammatory infiltrate.

The most effective treatment strategy includes targeted oral antibiotic therapy. Other treatment options could be considered for selected patients and include curettage, topical antibiotics, oral acitretin, intralesional, or systemic corticosteroids, and carbon dioxide laser debridement.^3^

## CONFLICT OF INTEREST

None declared.

## AUTHOR CONTRIBUTION

Noureddine Litaiem and Fatma Sahli wrote the first draft of the manuscript. Soumaya Gara, Olfa Charfi, and Soumaya Rammeh managed the literature searches and analyses. Mariem Jones and Faten Zeglaoui revised the manuscript. All the authors contributed to and have approved the final manuscript.

## ETHICAL APPROVAL

Approval of a review board was not required at our institution, in accordance with our country's law, because this study was a case report.

## CONSENT

Written informed consent was obtained from the patient to publish this report in accordance with the journal's patient consent policy.

## Data Availability

The data that support the findings of this article are available from the corresponding author upon reasonable request.
